# Sex differences in hepatic ischemia‒reperfusion injury: a cross-sectional study

**DOI:** 10.1038/s41598-023-32837-5

**Published:** 2023-04-07

**Authors:** Zhongyu Li, Shounan Lu, Baolin Qian, Zhanzhi Meng, Yongzhi Zhou, Dong Chen, Bangliang Chen, Guangchao Yang, Yong Ma

**Affiliations:** 1 Department of Minimal Invasive Hepatic Surgery, The First Affiliated Hospital of Harbin Medical University, Harbin, 150001 Heilongjiang China; 2 Key Laboratory of Hepatosplenic Surgery, Ministry of Education, Harbin, 150001 Heilongjiang China; 3 National Cancer Center/National Clinical Research Center for Cancer/Cancer Hospital & Shenzhen Hospital, Chinese Academy of Medical Sciences and Peking Union Medical College, Shenzhen, 518116 Guangdong China

**Keywords:** Hepatology, Liver diseases

## Abstract

Several studies have shown that males suffer more severe damage than females in the process of ischemia and reperfusion of the brain, heart and kidney. Accordingly, our study will reveal the correlation between the severity of hepatic ischemia‒reperfusion injury (HIRI) and sex, and preliminarily analyze the underlying mechanism. A total of 75 patients who were considered to have "benign liver tumors" at the initial admission and underwent partial hepatectomy were enrolled. We identified potential differences between different groups and discussed the correlation between the severity of HIRI and sex through a comparative analysis. Results showed that HIRI was more severe in males than in females, especially in younger patients. To explore whether estrogen level differences are the main reason for the sex differences in HIRI, we further revealed that HIRI in premenopausal females was more severe than that in postmenopausal females. By comparing the levels of gonadal hormones, we speculated that multiple gonadal hormones, including follicle-stimulating hormone, luteinizing hormone and testosterone, may jointly participate in the regulation of sex differences in HIRI together with estrogen.

## Introduction

Partial hepatectomy is the preferred treatment for various liver diseases, including hepatocellular carcinoma, cholangiocarcinoma, hepatic hemangioma, and focal nodular hyperplasia of the liver^1–3^. In recent years, the surgical technique of partial hepatectomy has developed rapidly, and breakthroughs have been made in many key links. However, hepatic damage caused by intraoperative ischemia‒reperfusion remains an important factor affecting the postoperative recovery of patients^4,5^. The occurrence of HIRI involves a variety of mechanisms, such as anaerobic injury, calcium overload, oxidative stress, microcirculation disturbance, sterile inflammatory response, apoptosis and autophagy, and the severity of injury is often related to the reserve function of the liver and intraoperative ischemic time^6–10^. Current studies have found sex differences in the incidence and progression of many diseases, such as Alzheimer's disease, gallbladder cancer, and nonalcoholic fatty liver disease, including ischemia‒reperfusion injury^11–16^. At present, studies on sex differences in ischemia‒reperfusion injury have mainly focused on ischemia‒reperfusion injury in the brain, heart and kidney, and most studies have shown that males suffer more severe damage than females during ischemia‒reperfusion in these organs^17–19^. Additionally, estrogen preconditioning has been reported to downregulate the expression of Ang II/AT1R in the process of HIRI and significantly reduce oxidative stress, while recruiting large numbers of infiltrating inflammatory cells, thereby reducing HIRI^20^. Moreover, studies have also suggested that the inhibition of estrogen sulfotransferase to reduce estrogen inactivation can also reduce HIRI^21^. However, conclusions on sex differences in HIRI are lacking, and few relevant clinical studies are available.

To explore the correlation between the severity of HIRI and sex, we selected patients diagnosed with "benign liver tumors" who underwent partial hepatectomy as the research subjects and divided all the enrolled patients into a male group and a female group. Because estrogen can be a protective factor in HIRI and female estrogen levels change significantly after menopause^22^, the female group was further divided into a premenopausal group and a postmenopausal group. Since the mean age of final menstrual period is around 50 years old^22^, the male group was correspondingly divided into a younger male group (age under 50 years old) and an elder male group (age at or above 50 years old) for stratified comparisons. In addition to liver function, coagulation function is also an important indicator of liver function in clinical practice. Therefore, we chose the prothrombin time (PT), prothrombin time activity (PTA), and international normalized ratio (INR) to compare the differences in coagulation function. In addition, alanine aminotransferase (ALT), aspartate aminotransferase (AST) and total bilirubin (TBil) are used to reflect changes in liver function. Through a comparative analysis of the above test results, the correlation between the severity of HIRI and sex was revealed, and the possible mechanism of the difference was preliminarily analyzed.

## Materials and methods

### Subjects and data collection

A total of 110 patients admitted to our hospital from September 2020 to January 2022 who were considered to have "benign liver tumors" were enrolled. The patients’ basic information, preoperative auxiliary examination results and intraoperative conditions were recorded in detail. We monitored their postoperative laboratory examination results, observed whether the patients had complications (e.g. bleeding, infection, bile leakage, and perihepatic effusion), and recorded the final pathological results. Based on the inclusion and exclusion criteria, 75 eligible cases were screened. All enrolled patients were divided into a male group with 21 cases and a female group with 54 cases according to sex. The female group was further divided into a premenopausal group with 31 cases and a postmenopausal group with 23 cases. The male group was divided into a younger male group with 9 cases and an elder male group with 12 cases.

### Inclusion and exclusion criteria

The inclusion criteria were as follows: (1) aged 18–75 years old, with no history of liver surgery; (2) Child‒Pugh grade A; (3) preoperative indocyanine green (ICG) 15-min retention rate < 10%; (4) first hepatic portal occlusion performed with a cycle of "blocking for 15 min and releasing the blocking for 5 min" during the operation; (5) postoperative pathology confirmed as benign liver tumor; (6) no other important organ diseases; and (7) complete and reliable clinical data.

The exclusion criteria were as follows: (1) combined with chronic hepatitis or liver cirrhosis; (2) significantly abnormal liver function before surgery, and liver-protecting drugs should be used; (3) anesthesia grade exceeding grade III; (4) important blood vessel or bile duct injury occurring during the operation; and (5) complications, such as bleeding, infection, bile leakage, and perihepatic effusion, occurring after the operation.

### Research preparation

Patients admitted to our hospital from September 2020 to January 2022 and considered to have "benign liver tumors" at the initial admission diagnosis were interviewed for general conditions, and the eligible patients were informed of the specific process and purpose of this study. The patients expressed informed consent and voluntarily cooperated with the collation and collection of clinical data and postoperative laboratory examination results. All procedures were approved by the Ethics Committee of the First Affiliated Hospital of Harbin Medical University.

After informed consent was obtained, the patients’ basic information (including name, sex and age) was collected. Preoperative laboratory examinations (including blood routine, coagulation function, liver function, renal function, and ion tests as well as hepatitis serial quantification and test of tumor biomarkers of common digestive system diseases) were performed for all patients. In addition, electrocardiography, echocardiography and lung CT were performed to evaluate the cardiopulmonary function of patients, and color Doppler ultrasonography of the liver, gallbladder, spleen and pancreas was also performed to initially evaluate the nature, size and number of tumors and the degree of liver steatosis. Three-phase contrast-enhanced CT or contrast-enhanced MRI of the liver was used to further clarify the location and nature of the tumor. According to the preoperative auxiliary results, the patient’s suitability and tolerance for partial hepatectomy were assessed. For patients who plan to undergo partial hepatectomy, ICG hepatobiliary dynamic imaging was further performed to evaluate the patient's liver reserve function, while the three-dimensional reconstruction of the liver showed the tumor size, location and relationship with surrounding blood vessels more clearly. During the operation, the central venous pressure (CVP) was measured every 10 min, and the average intraoperative CVP was calculated. The anesthesia grade, anesthesia time, operation time, times of Pringle maneuver, blood loss and blood transfusion condition were recorded. We monitored the patients’ postoperative blood routine, coagulation function, liver function, renal function and ion results, observed whether the patients had complications (e.g. bleeding, infection, bile leakage, and perihepatic effusion), and focused on the final pathological results to confirm whether the tumor was benign. Additionally, the volume of the partially resected liver was recorded.

### Observation indicator

1. The following indicators of patients were compared between different groups: general conditions: age, body mass index (BMI), albumin (ALB) level, degree of liver steatosis, and the size and number of tumors; preoperative levels of research indicators: PT, PTA, INR, ALT, AST and TBil; surgery-related factors: anesthesia time, operation time, average intraoperative CVP, times of Pringle maneuver, volume of the resected part of the liver, blood loss and blood transfusion condition.
2. The levels of PT, PTA, INR, ALT, AST, and TBil were monitored after the operation and compared between groups to evaluate the differences in the severity of HIRI.

### Statistical analysis

All data collation, comparison and statistical analysis in this study were carried out using SPSS 24.0 software. First, we analyzed the distribution of measurement data in each group. If the data obeyed the normal distribution, the mean ± standard deviation was used to describe, and two independent samples t test was used for intergroup comparisons; if the data did not obey the normal distribution, the median (interquartile range) was used to describe the data, and nonparametric test (Mann‒Whitney U test) was used for intergroup comparisons. The enumeration data are described with frequency (percentage), and the difference between the two groups was compared using the chi-square test. When comparing the enumeration data between the elder male group and the postmenopausal group, the sum of samples was less than 40, so we used Fisher's exact probability method for comparison. Variables were considered significantly different between groups when P < 0.05 (*P < 0.05; **P < 0.01; ***P < 0.001). GraphPad Prism 8.0 software was used to draw the statistical results.

### Ethics declarations

All procedures performed in studies involving human participants were following the ethical standards of the institutional and national research committee and with the 1964 Helsinki declaration and its later amendments or comparable ethical standards. Informed consent was obtained from all participants included in the study.

## Results

### HIRI was more severe in males than in females, especially in younger patients

As we know, surgery-related factors such as operation time and ischemia time may affect the severity of HIRI^23^. In addition, patients’ nutritional status, tumor-related factors and preoperative liver function level may affect our results as well. Therefore, to exclude confounding, we compared clinical characteristics (including general conditions, baseline laboratory data and intraoperative variables) between each pair of comparable groups. Through comparison, we found that there were no significant differences in these clinical characteristics between each pair of comparable groups (Supplementary Tables S1–S3).

**Figure 1:**
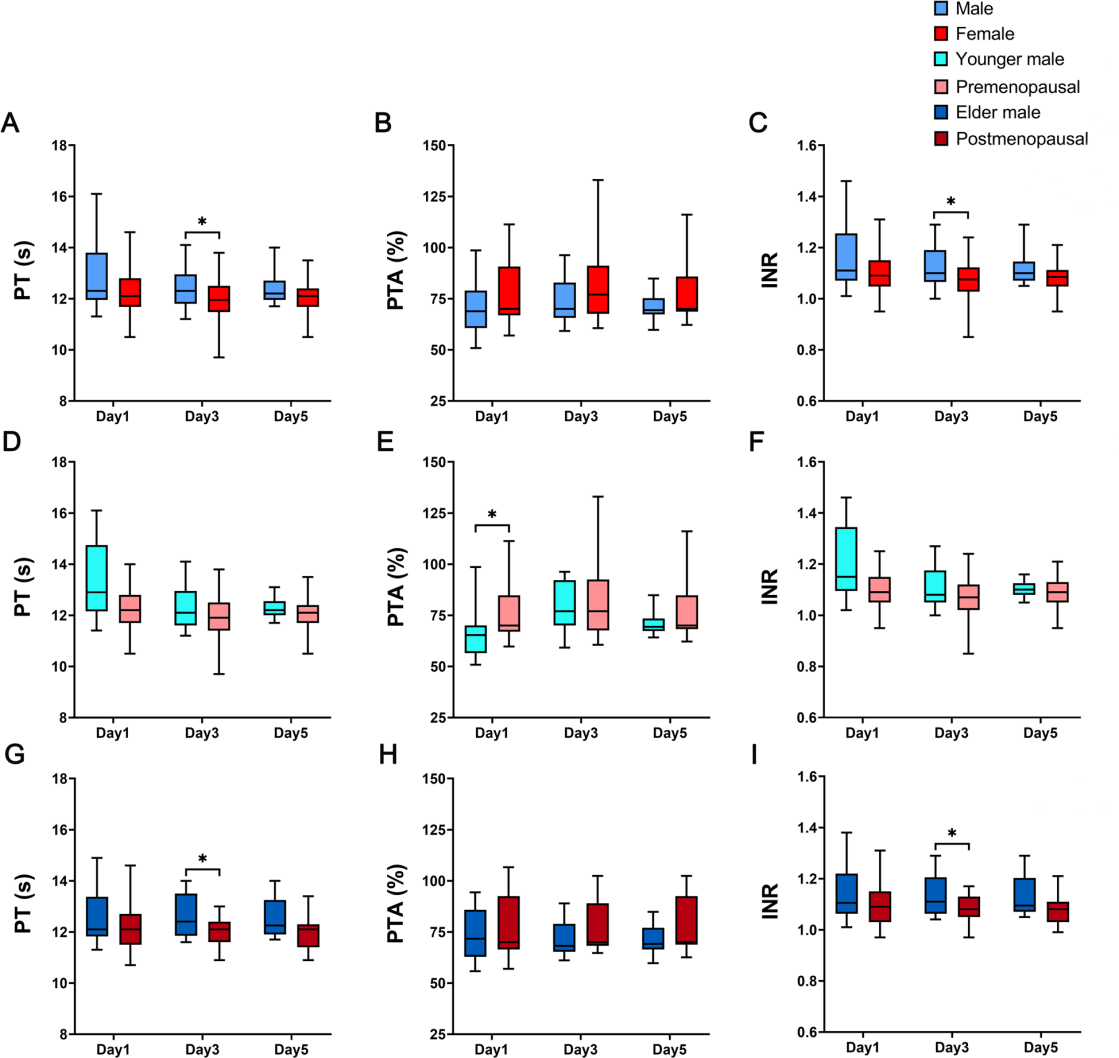
Comparison of coagulation function. (**A**) PT between male group and female group. (**B**) PTA between male group and female group. (**C**) INR between male group and female group. (**D**) PT between younger male group and premenopausal group. (**E**) PTA between younger male group and premenopausal group. (**F**) INR between younger male group and premenopausal group. (**G**) PT between elder male group and postmenopausal group. (**H**) PTA between elder male group and postmenopausal group. (**I**) INR between elder male group and postmenopausal group (*P < 0.05, **P < 0.01, ***P < 0.001).

First, we compared the changes in postoperative coagulation function between males and females. Through comparison, it was found that although there were slight differences in postoperative coagulation function between male patients and female patients, the results of PT, PTA and INR at all time points were within the normal range. Therefore, we suggest that the changes in coagulation function did not significantly differ between males and females in HIRI (Fig. 1).

**Figure 2:**
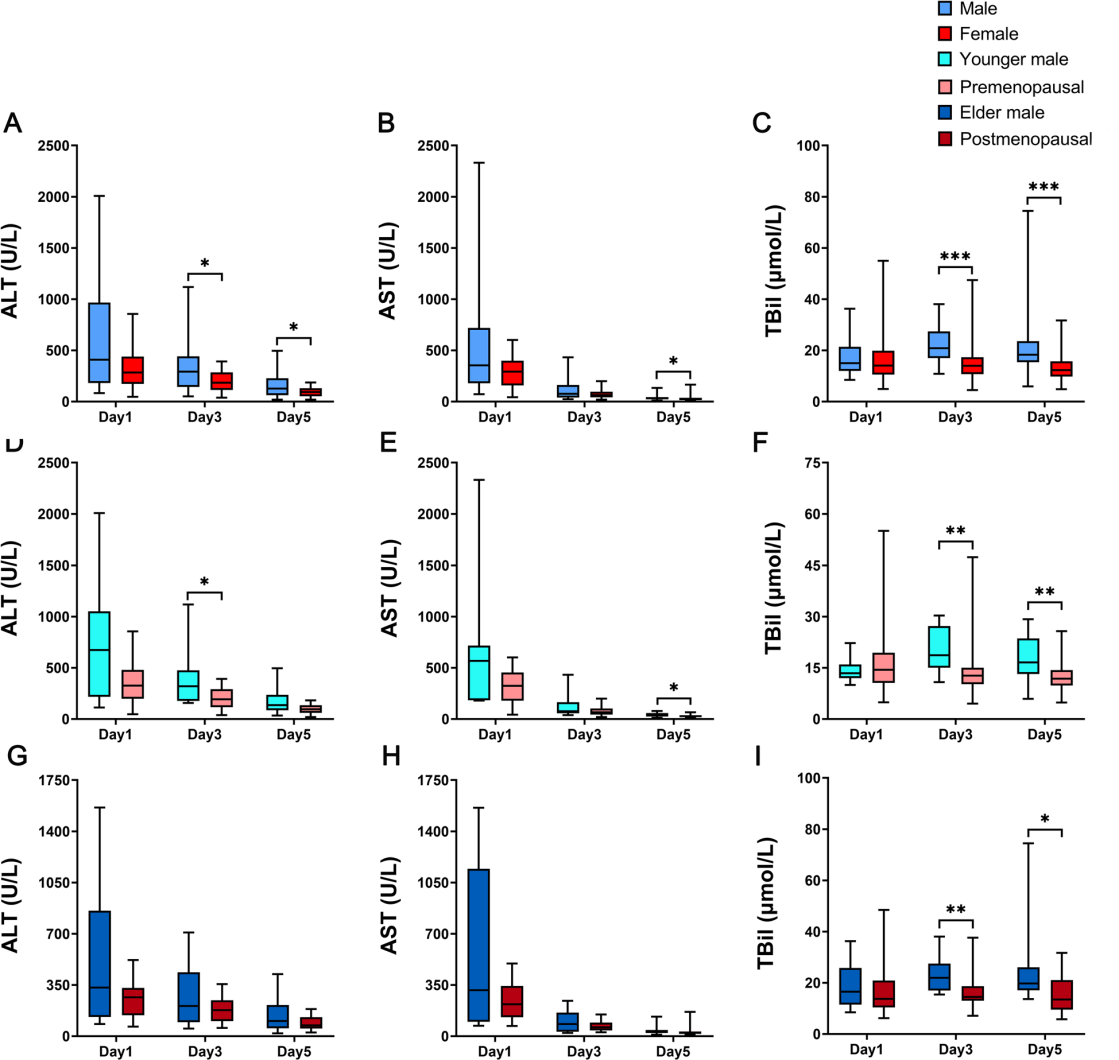
Comparison of liver function. (**A**) ALT between male group and female group. (**B**) AST between male group and female group. (**C**) TBil between male group and female group. (**D**) ALT between younger male group and premenopausal group. (**E**) AST between younger male group and premenopausal group. (**F**) TBil between younger male group and premenopausal group. (**G**) ALT between elder male group and postmenopausal group. (**H**) AST between elder male group and postmenopausal group. (**I**) TBil between elder male group and postmenopausal group (*P < 0.05, **P < 0.01, ***P < 0.001).

Next, we compared postoperative liver function levels between males and females. The results showed that male patients had significantly higher ALT levels than female patients after surgery, and AST levels on the first postoperative day were also significantly higher than those in female patients. The levels of TBil in male patients and female patients were both within the normal range, but were slightly higher in males than in females (Fig. 2).

According to the above results, we suggest that HIRI was more severe in males than in females. In addition, further stratified comparisons showed that this phenomenon is more pronounced in younger patients.

### HIRI was more severe in premenopausal females than in postmenopausal females

Based on a previous study reporting that estrogen can alleviate HIRI^20^, we further compared the premenopausal group with the postmenopausal group to assess whether different estrogen levels are the main reason for the sex differences in HIRI.

**Figure 3:**
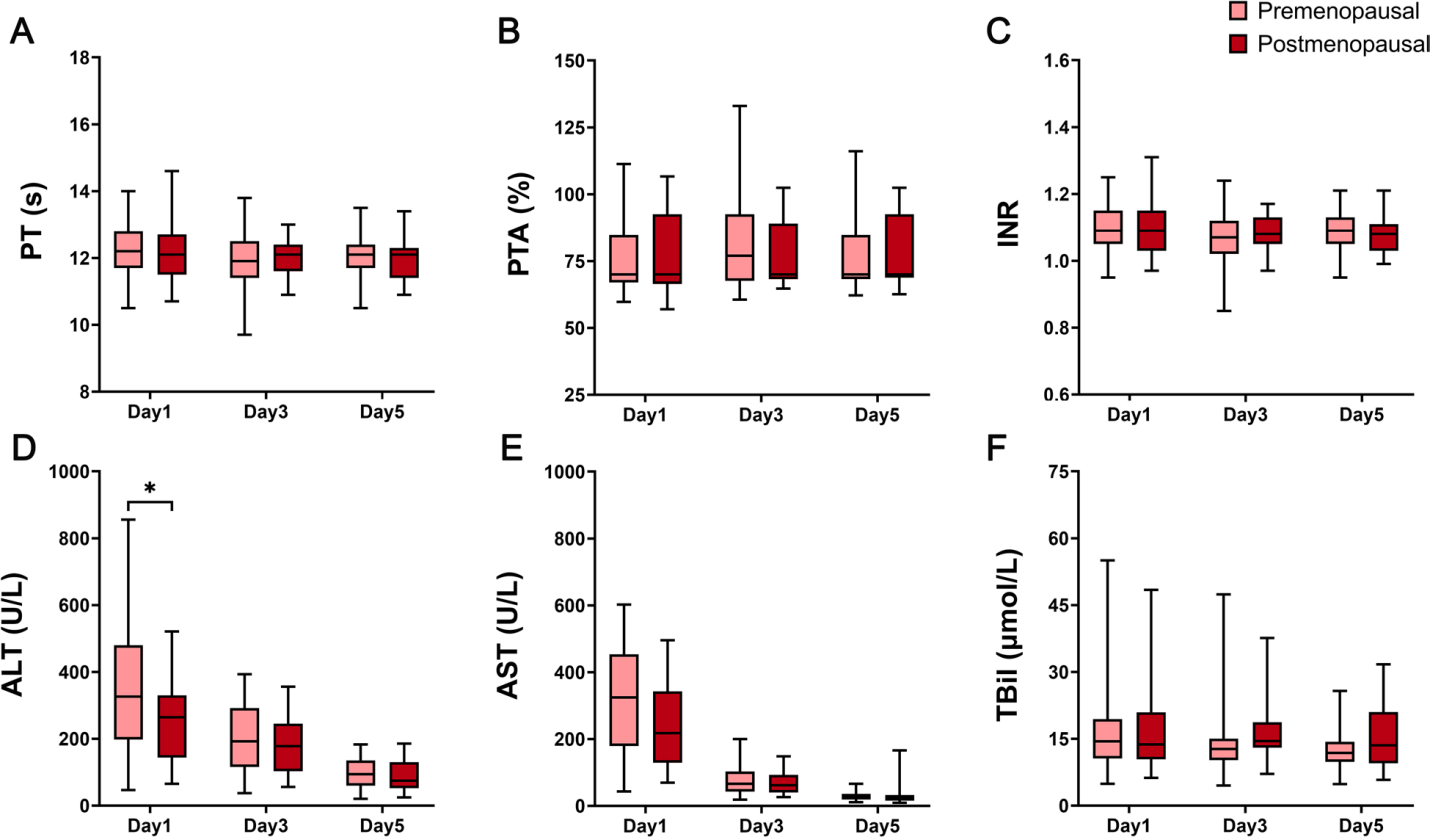
Comparison of coagulation function and liver function between premenopausal group and postmenopausal group. (**A**) PT, (**B**) PTA, (**C**) INR, (**D**) ALT, (**E**) AST, (**F**) TBil (*P < 0.05, **P < 0.01, ***P < 0.001).

Through comparison, we found no significant differences in clinical characteristics between the two groups except for age (Supplementary Table S4). We then compared postoperative coagulation function and liver function and found that coagulation function did not significantly differ between premenopausal females and postmenopausal females. However, the ALT and AST levels on the first postoperative day were significantly higher in premenopausal females than postmenopausal females, and the difference in ALT levels was statistically significant (Fig. 3).

In terms of the results, we suggest that HIRI was more severe in premenopausal females than in postmenopausal females, which is not consistent with the trend of estrogen changes between premenopausal and postmenopausal females. Therefore, we speculate that in addition to estrogen, other gonadal hormones may be involved in the regulation of HIRI.

### Multiple gonadal hormones may jointly participate in the regulation of sex differences in HIRI together with estrogen

To further explore the possibility of other gonadal hormones jointly regulating HIRI with estrogen, and to screen the target hormones, we first randomly selected 20 healthy premenopausal females and 20 healthy postmenopausal females to compare the levels of gonadal hormones (including progesterone (P), estradiol (E2), testosterone (T), follicle-stimulating hormone (FSH), luteinizing hormone (LH) and prolactin (PRL)). The comparison results showed that E2 level among premenopausal females was higher than postmenopausal females, while the levels of FSH and LH among premenopausal females were lower than postmenopausal females (Table 1). Therefore, we speculate that FSH and LH may play similar protective roles to E2.

**Table 1:** Comparison of gonadal hormones between premenopausal females and postmenopausal females.

Indicators	Premenopausal females (N = 20)	Postmenopausal females (N = 20)	*χ*^*2*^/t/z	*P* values
Age (years)	32.05 ± 2.87	59.15 ± 6.77	− 13.047	< 0.001***
P (ng/ml)	0.10 (0.10)	0.10 (0.08)	− 1.851	0.091
E2 (pg/ml)	30.50 (17.50)	7.50 (3.98)	− 5.311	< 0.001***
T (ng/dl)	33.46 (18.13)	29.85 (16.73)	− 0.717	0.478
FSH (mlu/ml)	5.20 (1.28)	41.20 (40.30)	5.412	< 0.001***
LH (mlu/ml)	4.30 (4.03)	18.85 (17.00)	5.248	< 0.001***
PRL (ng/ml)	11.80 (8.20)	10.80 (10.45)	− 0.595	0.565

*P < 0.05, **P < 0.01, ***P < 0.001.

We further compared the levels of gonadal hormones between males and females to explain the results of more severe HIRI in males than females. 20 healthy younger males (age under 50 years old) and 20 healthy elder males (age at or above 50 years old) were randomly selected and compared with premenopausal females and postmenopausal females, respectively. The comparison results showed that T level was significantly higher in males than females, while E2 level among premenopausal females was higher than younger males. Although E2 level of postmenopausal females was lower than that of elder males, FSH and LH levels were higher than those of elder males (Tables 2 and 3). We found that E2, FSH and LH play protective roles in HIRI could explain our results. However, T became another suspect variable.

**Table 2:** Comparison of gonadal hormones between younger males and premenopausal females.

Indicators	Younger males (N = 20)	Premenopausal females (N = 20)	*χ*^*2*^/t/z	*P* values
Age (years)	34.10 ± 8.07	32.05 ± 2.87	− 0.892	0.378
P (ng/ml)	0.10 (0.10)	0.10 (0.10)	− 1.242	0.253
E2 (pg/ml)	18.50 (8.75)	30.50 (17.50)	− 3.399	< 0.001***
T (ng/dl)	420.95 (192.58)	33.46 (18.13)	− 15.394	< 0.001***
FSH (mlu/ml)	4.55 (2.73)	5.20 (1.28)	− 1.476	0.142
LH (mlu/ml)	3.15 (1.85)	4.30 (4.03)	1.673	0.105
PRL (ng/ml)	10.40 (5.35)	11.80 (8.20)	− 1.475	0.142

*P < 0.05, **P < 0.01, ***P < 0.001.

**Table 3:** Comparison of gonadal hormones between elder males and postmenopausal females.

Indicators	Elder males (N = 20)	Postmenopausal females (N = 20)	*χ*^*2*^/t/z	*P* values
Age (years)	57.10 ± 5.14	59.15 ± 6.77	1.023	0.313
P (ng/ml)	0.10 (0.12)	0.10 (0.08)	− 0.029	0.989
E2 (pg/ml)	20.50 (14.50)	7.50 (3.98)	3.963	< 0.001***
T (ng/dl)	482.15 (276.01)	29.85 (16.73)	5.410	< 0.001***
FSH (mlu/ml)	4.70 (6.13)	41.20 (40.30)	− 5.357	< 0.001***
LH (mlu/ml)	3.90 (2.83)	18.85 (17.00)	− 5.356	< 0.001***
PRL (ng/ml)	8.15 (5.48)	10.80 (10.45)	− 1.028	0.314

*P < 0.05, **P < 0.01, ***P < 0.001.

**Figure 4:**
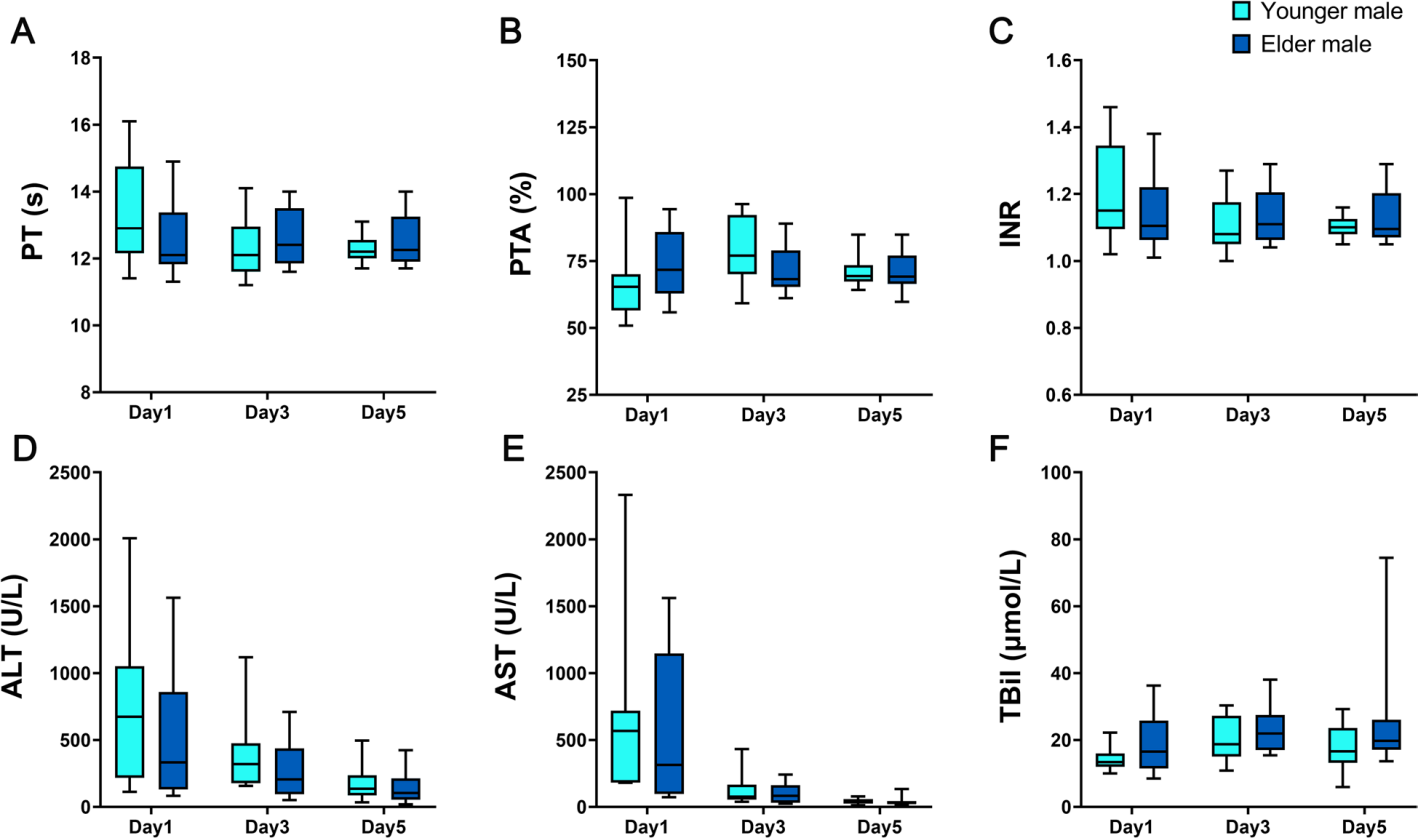
Comparison of coagulation function and liver function between younger male group and elder male group. (**A**) PT, (**B**) PTA, (**C**) INR, (**D**) ALT, (**E**) AST, (**F**) TBil (*P < 0.05, **P < 0.01, ***P < 0.001).

For further exploration, we compared the hormone levels between healthy younger males and elder males, and the severity of HIRI between the younger male group patients and elder male group patients. It was found that there were no significant differences in the severity of HIRI between the two patient groups. Also, there were no significant differences in the levels of gonadal hormones between healthy younger males and elder males (Fig. 4 and Table 4). Therefore, we cannot rule out the possibility that T is related to the mechanism of aggravating the injury in HIRI.

**Table 4:** Comparison of gonadal hormones between younger males and elder males.

Indicators	Younger males (N = 20)	Elder males (N = 20)	*χ*^*2*^/t/z	*P* values
Age (years)	34.10 ± 8.07	57.10 ± 5.14	− 10.774	< 0.001***
P (ng/ml)	0.10 (0.10)	0.10 (0.12)	− 0.157	0.883
E2 (pg/ml)	18.50 (8.75)	20.50 (14.50)	− 0.271	0.799
T (ng/dl)	420.95 (192.58)	482.15 (276.01)	− 0.367	0.716
FSH (mlu/ml)	4.55 (2.73)	4.70 (6.13)	1.218	0.231
LH (mlu/ml)	3.15 (1.85)	3.90 (2.83)	− 1.470	0.150
PRL (ng/ml)	10.40 (5.35)	8.15 (5.48)	0.674	0.504

*P < 0.05, **P < 0.01, ***P < 0.001.

Based on the above results, we suggest that multiple gonadal hormones, including FSH, LH and T, may jointly participate in the regulation of sex differences in HIRI together with estrogen.

## Discussion

HIRI is the main cause of liver function damage and even liver failure after partial hepatectomy, which seriously affects the development of liver surgery^24^. In-depth studies identified sex differences in the severity of ischemia–reperfusion injury in many organs^17–19^. However, it is still inconclusive on whether there are sex differences in HIRI, and relevant clinical studies are scarce. Potential sex differences in HIRI would have important guiding significance for the clinical diagnosis and treatment of patients undergoing partial hepatectomy. Therefore, we carried out this clinical study to reveal the correlation between the severity of HIRI and sex and to preliminarily analyze the possible mechanisms.

To avoid the possible impact on the postoperative liver function of patients, we excluded patients with hepatocellular carcinoma, cholangiocarcinoma, metastatic liver cancer, hepatic cystadenocarcinoma and other malignant liver diseases. Only patients with "benign liver tumors" who underwent partial hepatectomy were included in the study. Furthermore, considering that current studies have confirmed that estrogen can play a protective role in HIRI^20,21^, we speculate that estrogen may be the main factor causing sex differences in HIRI. Because the estrogen level of females changes greatly after menopause, females over the age of 40 who did not have a period for 12 months after the last menstrual period can be clinically diagnosed as being menopausal when pregnancy is excluded^25^. Thus, the female group was further divided into a premenopausal group and a postmenopausal group, and the male group was correspondingly divided into a younger male group (age under 50 years old) and an elder male group (age at or above 50 years old) for stratified comparisons according to the mean age of females’ final menstrual period is around 50 years old^22^.

Since the important coagulation factors II, VII and X involved in the extrinsic coagulation pathway are all synthesized by hepatocytes^26^, PT will be prolonged in the early stage of hepatocyte damage, and PT prolongation will increase as hepatocyte damage worsens^27^. PTA can more accurately reflect the activity of prothrombin, and INR can reflect the ratio of prothrombin time to normal control prothrombin time. Therefore, we also selected PT, PTA and INR as observation indicators in addition to ALT, AST and TBil.

In partial hepatectomy, there are some surgery-related factors that can affect the severity of HIRI. In addition, patients’ nutritional status, tumor-related factors and preoperative liver function level may affect our results as well. Therefore, we compared whether there were differences in clinical characteristics (including general conditions, baseline laboratory data and intraoperative variables) between each pair of comparable groups at the beginning. Through a comprehensive analysis of our clinical data, we have confirmed that there are indeed sex differences in HIRI, and HIRI is more severe in males than in females. In females, HIRI is more severe in premenopausal females than in postmenopausal females. However, there were no differences in clinical characteristics between each pair of comparable groups. Therefore, we supposed that although these clinical characteristics may indeed affect the severity of HIRI between individuals, they were not the cause of sex differences in HIRI.

A previous study also suggested that the sex differences in HIRI are related to the sex-determining region on the Y chromosome^28^, which cannot fully explain the results of our study. Therefore, we supposed that in addition to the sex-determining region on the Y chromosome, the differences in gonadal hormone levels between males and females may also explain the sex differences in HIRI. After further comparing the hormone levels of males and females in different periods, we suggest that multiple gonadal hormones, including FSH, LH and T, may jointly participate in the regulation of sex differences in HIRI together with estrogen.

FSH is secreted by the pituitary gland and plays a vital role in the regulation of reproductive function in both males and females. The current study found that many other organs can express FSH, such as the stomach, and the function of FSH is also beyond the regulation of reproduction. It is reported that FSH can reduce the apoptosis of neurons and alleviate ischemic injury by downregulating the expression of Fas^29^. The Fas/Fasl pathway is also one of the important pathways in ischemia–reperfusion injury. In addition, FSH can also regulate the expression of p53 upregulated modulator of apoptosis to inhibit oxidative stress-induced apoptosis through the PI3K/Akt pathway^30^. Therefore, we believe that FSH may also play a protective role and act as one of the factors that cause sex differences in HIRI. Regarding the role of T in ischemia–reperfusion injury, most of the previous studies were conducted in cardiac ischemia–reperfusion injury and renal ischemia–reperfusion injury. Some studies have suggested that T can promote inflammation by increasing the activation of JNK and p38, and apoptosis by upregulating Fas and decreasing Bcl-2 while increasing Bax expression^31^. Therefore, it is considered that T can aggravate ischemia–reperfusion injury through pro-inflammatory and pro-apoptotic effects^32,33^, while T deficiency can alleviate ischemia–reperfusion injury^34,35^. However, there are also opposing views that T plays a protective role in ischemia-reperfusion^36,37^. According to our results, we suggest that T may aggravate the injury in HIRI. As for LH, we found no literature has reported that there is a relation between LH and mechanisms of ischemia–reperfusion injury. In summary, we supposed that sex differences in HIRI may be related to the different expression levels of E2, FSH and T between males and females. Moreover, E2 and FSH can alleviate the injury, while T is related to the mechanism of aggravating the injury. To verify the specific relationship between gonadal hormone levels (especially E2, FSH and T) and the severity of HIRI, we plan to further detect patients’ gonadal hormones levels in future studies and conduct correlation analysis between the severity of HIRI and these gonadal hormone levels. We also plan to treat animal models and primary hepatocytes with exogenous hormones to explore the possible related mechanisms.

In addition, other biochemical and molecular factors that differ between males and females may also contribute to sex differences in HIRI. Previous studies have suggested that there are sex differences in the expression of inflammatory cytokines such as HSP27 and MCP-1, which may lead to sex differences in ischemia–reperfusion injury^19,38^. Estrogen and estrogen receptor are involved in the regulation of mitochondrial permeability transitions (MPT), and sex differences in MPT can cause differences in the severity of calcium overload, leading to sex differences in ischemia–reperfusion injury^17^. In addition, autophagy is one of the mechanisms of HIRI, while X chromosome, androgen receptor and estrogen receptor are involved in the regulation of autophagy, which may also contribute to the sex differences in HIRI^39^. These factors should also be brought to our attention.

In our study, all patients underwent laparoscopic partial hepatectomy by the same surgeon, and all patients received prophylactic analgesia after surgery. To ensure clinical safety and ethical considerations, all patients needed to receive postoperative liver protection treatment, which may affect the severity of HIRI^40,41^. In addition, because some studies indicated that commonly used surgical anesthesia drugs, such as sevoflurane and isoflurane, have a protective effect on HIRI^42,43^, we also strive to use the same anesthesia plan for all patients during surgery. However, since clinical safety is a priority, small differences sometimes cannot be avoided. Due to the impact of the COVID-19 epidemic, our study has a limited clinical sample size. In subsequent studies, we will continue to expand the sample size to improve the representativeness of the research results.

## Supplementary Information

Supplementary Information.

## Data Availability

The data that support the findings of this study are available from the corresponding author upon reasonable request.
