# Supplementary material for: Sex differences in hepatic ischemia‒reperfusion injury: a cross-sectional study
Source: Sci Rep. 2023 Apr 7;13:5724. doi: 10.1038/s41598-023-32837-5 (PMC10081297; doi:10.1038/s41598-023-32837-5)
Supplement: Supplementary file 1 — Supplementary Information. [file 41598_2023_32837_MOESM1_ESM.pdf]

Supplementary data

Table S1. Comparison of general conditions.

	Age (years)	BMI (Kg/m2)	ALB (g/L)	Fatty liver		Tumor diameter (cm)	Number of tumor	
				mild	N/A		Single	Multiple
Male group (N=21)	51.00 (12.00)	24.41 ± 2.45	40.67 ± 2.89	8 (38.10)	13 (61.90)	7.10 (5.00)	11 (52.38)	10 (47.62)
Female group (N=54)	48.50 (12.50)	23.95 ± 2.97	40.56 ± 2.93	16 (29.63)	38 (70.37)	7.50 (2.55)	27 (50.00)	27 (50.00)
χ^2/t/z	0.107	0.636	0.145	0.498		-0.053	0.034	
P values	0.915	0.527	0.885	0.480		0.958	0.853	
Younger male group (N=9)	42.00 (13.00)	25.05 ± 2.95	41.58 ± 1.42	5 (55.56)	4 (44.44)	9.00 (4.35)	4 (44.44)	5 (55.56)
Premenopausal group (N=31)	43.00 (9.00)	24.18 ± 2.87	40.26 ± 2.57	8 (25.81)	23 (74.19)	7.30 (2.30)	15 (48.39)	16 (51.61))
χ^2/t/z	0.745	0.800	1.458	1.621		1.925	0.000	
P values	0.750	0.429	0.153	0.203		0.062	1.000	
Elder male group (N=12)	54.00 (7.00)	23.93 ± 2.00	39.98 ± 3.54	3 (25.00)	9 (75.00)	5.85 (4.13)	7 (58.33)	5 (41.67)
Postmenopausal group (N=23)	56.00 (6.00)	23.63 ± 3.14	40.95 ± 3.38	8 (34.78)	15 (65.22)	7.60 (2.80)	12 (52.17)	11 (47.83)
χ^2/t/z	0.401	0.297	-0.792			-1.181		
P values	0.420	0.769	0.434	0.709		0.246	1.000	

*$P<0.05$, **$P<0.01$, ***$P<0.001$

Table S2. Comparison of Baseline laboratory data

	PT (s)	PTA (%)	INR	A LT (U/L)	AST (U/L)	TBil (μmol/L)
Male group (N=21)	11.10 (1.50)	98.30 (24.45)	0.99 (0.13)	18.30 (7.05)	16.80 (7.00)	12.80 (4.90)
Female group (N=54)	11.30 (0.83)	94.40 (19.63)	1.02 (0.09)	16.10 (5.25)	15.55 (3.35)	10.75 (5.98)
χ^2/t/z	0.455	0.537	0.479	-1.670	-0.749	-1.688
P values	0.649	0.593	0.632	0.095	0.454	0.091
Younger male group (N=9)	10.90 (1.65)	102.40 (34.55)	0.99 (0.15)	18.70 (6.55)	16.80 (8.55)	12.80 (6.15)
Premenopausal group (N=31)	11.40 (0.80)	94.40 (19.10)	1.03 (0.08)	15.70 (5.10)	14.90 (3.40)	9.50 (6.50)
χ^2/t/z	-0.940	0.983	-1.108	0.060	0.604	0.646
P values	0.353	0.332	0.275	0.059	0.610	0.522
Elder male group (N=12)	11.40 (1.53)	92.50 (23.83)	1.03 (0.12)	17.85 (7.78)	16.80 (6.75)	12.55 (4.25)
Postmenopausal group (N=23)	11.00 (1.00)	96.30 (23.40)	1.01 (0.10)	16.40 (13.00)	16.30 (3.30)	11.20 (8.90)
χ^2/t/z	0.590	-0.166	0.554	0.754	0.702	0.348
P values	0.595	0.869	0.572	0.771	0.719	0.362

$^*P<0.05$, $^{**}P<0.01$, $^{***}P<0.001$

Table S3. Comparison of Intraoperative variables

	Anesthesia time (min)	Operation time (min)	Mean CVP (cmH2O)	Times of Pringle's (times)	Resection volume (cm3)	Blood loss (mL)	Transfusion of blood	
							Yes	No
Male group (N=21)	285.00 (187.50)	210.00 (180.00)	2.50 (0.25)	3.00 (2.50)	175.00 (416.85)	50.00 (280.00)	9 (42.86)	12 (57.14)
Female group (N=54)	270.00 (97.50)	202.50 (97.50)	2.50 (0.30)	3.00 (2.00)	168.00 (219.50)	100.00 (150.00)	19 (35.19)	35 (64.81)
χ^2/t/z	0.839	-0.496	-1.641	-0.199	0.030	0.534	0.380	
P values	0.404	0.620	0.101	0.842	0.976	0.594	0.537	
Younger male group (N=9)	390.00 (282.50)	315.00 (212.50)	2.50 (0.40)	4.00 (2.00)	384.00 (421.50)	200.00 (365.00)	5 (55.56)	4 (44.44)
Premenopausal group (N=31)	280.00 (105.00)	195.00 (105.00)	2.50 (0.30)	3.00 (2.00)	168.00 (260.00)	100.00 (150.00)	12 (38.71)	19 (61.29)
χ^2/t/z	0.277	0.299	0.718	0.064	0.517	0.780	0.267	
P values	0.291	0.306	0.477	0.069	0.524	0.799	0.605	
Elder male group (N=12)	275.00 (120.00)	195.00 (120.00)	2.55 (0.18)	2.00 (2.00)	140.00 (417.67)	50.00 (80.00)	4 (33.33)	8 (66.67)
Postmenopausal group (N=23)	255.00 (110.00)	210.00 (90.00)	2.40 (0.40)	3.00 (1.00)	192.00 (205.13)	50.00 (50.00)	7 (30.43)	16 (69.57)
χ^2/t/z	-0.020	-0.243	1.597	0.236	0.476	0.532		
P values	0.984	0.809	0.120	0.263	0.482	0.548	1.000	

$^*P<0.05$, $^{**}P<0.01$, $^{***}P<0.001$

Table S4. Comparison of clinical characteristics between premenopausal group and postmenopausal group

Indicators		Premenopausal group (N=31)	Postmenopausal group (N=23)	χ^2/t/z	*P* values
**General conditions**					
Age (years)		43.00 (9.00)	56.00 (6.00)	6.243	<0.001***
BMI (Kg/m^2)		24.18 ± 2.87	23.63 ± 3.14	0.669	0.506
ALB (g/L)		40.26 ± 2.57	40.95 ± 3.38	-0.849	0.400
Fatty liver	Mild	8 (25.81)	8 (34.78)	0.510	0.475
	N/A	23 (74.19)	15 (65.22)		
Tumor diameter (cm)		7.30 (2.30)	7.60 (2.80)	-0.677	0.501
Number of tumor	Single	15 (48.39)	12 (52.17)	0.076	0.783
	Multiple	16 (51.61)	11 (47.83)		
**Baseline laboratory data**					
PT (s)		11.40 (0.80)	11.00 (1.00)	-0.999	0.318
PTA (%)		94.40 (19.10)	96.30 (23.40)	-0.441	0.661
INR		1.03 (0.08)	1.01 (0.10)	-0.815	0.415
ALT (U/L)		15.70 (5.10)	16.40 (13.00)	1.785	0.074
AST (U/L)		14.90 (3.40)	16.30 (3.30)	1.146	0.252
TBil (μmol/L)		9.50 (6.50)	11.20 (8.90)	1.207	0.227
**Intraoperative variables**					
Anesthesia time (min)		280.00 (105.00)	255.00 (110.00)	-0.937	0.349
Operation time (min)		195.00 (105.00)	210.00 (90.00)	-0.657	0.511
Mean CVP (cmH_2O)		2.50 (0.30)	2.40 (0.40)	0.876	0.385
Times of Pringle's (times)		3.00 (2.00)	3.00 (1.00)	-1.179	0.238
Resection volume (cm^3)		168.00 (260.00)	192.00 (205.13)	1.033	0.302
Blood loss (ml)		100.00 (150.00)	50.00 (50.00)	-1.851	0.064
Transfusion of blood	Yes	12 (38.71)	7 (30.43)	0.396	0.529
	No	19 (61.29)	16 (69.57)		

*P<0.05, **P<0.01, ***P<0.001
